# Measurable residual disease testing in acute myeloid leukemia: current state, foundational models, and tools for future development

**DOI:** 10.1007/s10555-026-10322-5

**Published:** 2026-03-27

**Authors:** Joseph Van Galen, Stephen D. Willis, Ashish Bains, Sara H. Small

**Affiliations:** 1 Division of Transplant and Cell Therapy, Department of Medical Oncology, Sidney Kimmel Medical College, Thomas Jefferson University, Philadelphia, PA USA; 2 Immune Cell Regulation and Targeting Program, Sidney Kimmel Comprehensive Cancer Center, Thomas Jefferson University, 834 Chestnut St, Ste 308, Philadelphia, PA 19107 USA; 3 Department of Bone Marrow Transplant and Cellular Therapies, Fox Chase Cancer Center at Temple Health, Philadelphia, PA USA; 4 Nuclear Dynamics and Cancer Program, Fox Chase Cancer Center at Temple Health, Philadelphia, PA USA; 5 Cancer Epigenetics Institute, Fox Chase Cancer Center at Temple Health, Philadelphia, PA USA; 6 Fels Cancer Institute for Personalized Medicine, Lewis Katz School of Medicine at Temple University, Pharmacy Allied Health Building, 3307 N. Broad St., Room 204, Philadelphia, PA 19140 USA; 7 Department of Pathology and Laboratory Medicine, Lewis Katz School of Medicine at Temple University, Philadelphia, PA USA

**Keywords:** Acute myeloid leukemia, Measurable residual disease, Personalized medicine, Next-generation sequencing, Clonal hematopoiesis of indeterminate potential, Hematopoiesis

## Abstract

Acute myeloid leukemia (AML) is a lethal and rapidly progressive hematologic malignancy with high rates of relapse and treatment refractoriness. Management of AML is complicated by biological heterogeneity in a disease that is broadly defined by the clonal expansion of myeloblasts that otherwise play an important role in healthy marrow tissues. While subtypes of AML are increasingly defined by druggable driver mutations including *FLT3-ITD*, *IDH1*, *IDH2*, and *NPM1*, conventional chemotherapy and reduced intensity induction regimens (e.g., azacitidine-venetoclax) remain therapeutic backbones. One area of active development for personalization of AML treatment is the assessment of measurable residual disease (MRD). MRD testing in AML is complicated by uncertainty regarding the physiologic compartment of persistent and relapsing myeloblasts, and by increasing recognition of myeloid driver mutations in some healthy bone marrow states, such as clonal hematopoiesis of indeterminate potential (CHIP). Even in large academic centers, MRD tools are not yet universally available. Standardized workflows for MRD implementation are only beginning to enter consensus and guideline documents. Current understanding of AML biology and state-of-the-art tools for MRD measurement are reviewed here in an effort to promote clinical and laboratory investigator collaboration for the development of reliable tools for improving outcomes in this deadly disease. Clinical trial number: not applicable

## Introduction

Acute myeloid leukemia (AML) is a rare but deadly hematologic malignancy that can present in three principal forms: (1) *de novo* disease, (2) as a complication of prior chemotherapy or radiation exposure, or (3) as a secondary malignancy that emerges from a precursor myeloid neoplasm. While the rate of subsequent mutagenesis after diagnosis is relatively low compared to other aggressive cancers, clonal or subclonal genetic or molecular aberrations are detectable in nearly all clinical cases at presentation [1, 2]. The myeloblasts whose clonal growth defines AML exist across a spectrum of genetic, cytogenetic, and immunophenotypic types. There has been a progression from subclassification based on microscopic features to disease definition and risk stratification based on molecular findings. Given constant improvement in the sensitivity of molecular tools available for clinical use, this shift has been accompanied by an increasing focus on investigating the significance of disease activities detectable only at very low burdens, especially after initial induction therapy.

In recent years, the monitoring of measurable residual disease (MRD) has become an area of intense study across multiple tumor types and has entered clinical management guidelines for a limited number of hematologic malignancies [3–5]. Historically, the same biologic finding was referred to as “minimal residual disease.” Both terms describe small burdens of disease that can be detected by sensitive molecular or other tools but remain below the threshold of conventional histopathologic assessment.

The heterogeneity of AML poses a fundamental challenge to the implementation of MRD-based treatment approaches. While pathognomonic surface or intracellular markers permit reliable detection of malignant cells at low frequencies in certain lymphoid malignancies, for example, no such universal markers exist in AML. Although molecular and immunophenotypic aberrations are frequently found, each is observed in only a subset of patients. The proliferating myeloblasts that define active AML are often phenotypically similar to normal bone marrow precursors, and many of the molecular markers used to identify AML activity may also be seen in pre- and non-malignant conditions such as clonal hematopoiesis of indeterminate potential (CHIP). Given these dynamics, the route to effective use of clinical MRD assays in AML management will likely involve validation of a range of individual tools, each in a specific clinical context, at least until next-generation understandings of AML pathogenesis and mechanisms of relapse are accepted.

The growing interest in MRD testing in AML is driven by its potential to improve patient stratification, refine prognosis, and guide therapy once conventional remission has been achieved. MRD monitoring has already transformed the management of acute lymphoblastic leukemia and multiple myeloma, where MRD negativity is recognized as an important predictor of improved survival [6–9]. Standardized workflows for MRD measurement in molecularly defined AML subtypes are only beginning to be incorporated into professional guideline documents for physicians, and access to commercial and laboratory-developed assays is unevenly distributed by geography and practice setting. Nevertheless, the promise of MRD-informed decision-making continues to grow as increasingly sophisticated genetic, epigenetic, cytogenetic, and flow-based tools transition from the research setting into the clinical pathology laboratory. One central evolution gaining momentum is the move away from bulk sample characterization and toward selected or even single-cell techniques that allow colocalization of multiple cell features to provide greater resolution of AML clonal architecture.

**Fig. 1:**
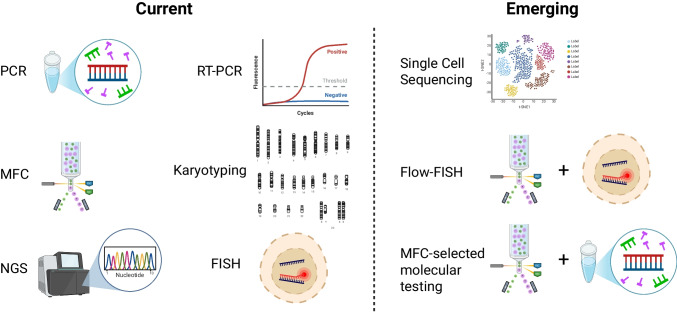
Emerging multimodal tools for measurable residual disease testing in acute myeloid leukemia. Created in BioRender. Small, S. (2025) https://BioRender.com/4shgmwp

The goal of this review is to support the development and adoption of MRD tools that can benefit a wider spectrum of AML patients. We begin by exploring AML as a clinical construct, detailing the biologic and clinicopathologic features necessary to understand MRD development. We summarize the state of the art with respect to MRD implementation in AML management, with a special focus on the various molecular platforms that can be used to measure MRD, each of which offers a unique mix of advantages and limitations. A summative discussion details the combined-modality tools that promise improved operating characteristics and potential availability of MRD testing for a larger group of AML patients in the near future (Fig. 1).

## Current paradigms of AML management

AML is a heterogeneous disease that, despite significant advances in the past decade, remains difficult to manage and even more difficult to cure, with an estimated 5-year overall survival rate of 32% [10]. Patients typically present to healthcare professionals with recurrent infections due to neutropenia, signs of leukostasis (due to hyperleukocytosis), fatigue, and shortness of breath due to anemia, and/or bleeding and bruising due to thrombocytopenia. They may have additional laboratory abnormalities due to spontaneous tumor lysis syndrome or disseminated intravascular coagulopathy in the case of acute promyelocytic leukemia (APL). In all cases, the disease must be diagnosed and managed quickly to prevent initial mortality due to complications of untreated disease. Initial diagnosis is typically made with a combination of immunophenotyping by flow cytometry and immunohistochemistry performed on peripheral blood and bone marrow specimens. Additional standard molecular studies include next-generation sequencing (NGS), cytogenetics, and fluorescence *in situ* hybridization (FISH). Whether and which results of these tests are usually available quickly enough to guide initial treatment choice will vary by practice setting. With the incorporation of these molecular tools, diagnostic criteria have moved away from only using the historic arbitrary cut-off of 20% myeloblasts as defining AML [11, 12]. In the modern era, the thresholds that define AML from precursor conditions vary among molecular subtypes.

Whether or not such results are immediately available, molecular analysis is essential for stratification of AML patients into risk categories (favorable, intermediate, or adverse risk) among which prognosis and upfront treatment approaches vary. For example, favorable risk disease can achieve long-term remission with chemotherapy alone while those with intermediate or adverse risk disease need an allogeneic stem cell transplant (allo-SCT).

Among the molecular variants that define favorable risk disease are (1) t(8;21), which produces the *RUNX1-RUNX1T1* transcript, (2) core-binding factor “mutations” (including inv(16) or t(16;16), both of which produce *CBFB-MYH11)*, (3) bZIP in-frame mutated *CEBPA*, and (4) mutated *NPM1*(without *FLT3-ITD*) [13]. Intermediate risk disease markers include *FLT3* internal tandem duplications (*FLT3-ITD*) and t(9;11), which produces the *MLLT3-KMT2A* transcript [12]. Adverse risk defining factors are most numerous, and include both molecular and clinical findings, among them complex karyotypes, deletions typically seen in MDS (−5, del(5q), −7, −17, abnormal 17p), *TP53* mutations, and splicing factor mutations (*SF3B1, SRSF2, ZRSR2, and U2AF1*) [13]. AML that arises from a preceding myelodysplastic syndrome (secondary AML) or that arises in a patient who was previously treated with chemotherapy (therapy-related AML) is considered adverse risk disease, as are cases that result in the formation of solid masses of AML cells (i.e., chloroma/myeloid sarcoma).

Induction therapy for newly diagnosed AML usually consists of intensive chemotherapy (a combination regimen including cytarabine and an anthracycline) or less intensive therapy, where a hypomethylating agent is administered in combination with the BCL-2 inhibitor venetoclax [14]. Treatment is selected based on patient factors such as age, fitness, comorbidities, and disease factors such as cytogenetics and molecular changes. Targeted agents that may be used in different combinations include the CD33 antibody-drug conjugate gemtuzumab-ozogamicin, *FLT3* inhibitors such as midostaurin, gilteritinib, or quizartinib, *IDH1* inhibitors such as ivosidenib or olutasidenib, the *IDH2* inhibitor enasidenib, or menin inhibitors such as revumenib for *NPM1-*mutated and *KMT2A*-rearranged disease: how to incorporate these agents in combination with or in lieu of conventional induction agents is an area of rapid exploration. After primary induction, disease response is determined by repeat bone marrow aspiration and biopsy.

If intensive chemotherapy was used for induction and a patient has achieved a morphologic complete remission (CR, defined by a hypocellular marrow with less than or equal to 5% residual blasts), patients are generally treated with up to four cycles of consolidation therapy with high dose cytarabine and/or allo-SCT. With intensive therapy, a CR can be achieved in 50–70% of patients [14]. Unfortunately, up to 80% of patients who achieve an initial CR will later go on to relapse [15, 16]. Median overall survival after relapse is less than 6 months [17]. Less intensive first-line therapies are continued until unacceptable toxicity or disease progression and are not expected to be curative unless coupled with allo-SCT.

For patients with a morphologic CR, current NCCN guidelines recommend MRD assessments for specific molecular subgroups at defined intervals: after the first cycle of consolidation chemotherapy followed by every 6–12 weeks for 2 years for *NPM1*-mutated, *CBFB-MYH11*, and *RUNX1-RUNX1T1* disease [14]. For patients with *NPM1*-mutated disease receiving lower-intensity therapy (e.g., hypomethylating agent with venetoclax), MRD assessment is recommended every 6–12 weeks.

As explored and explained in detail throughout this review, how to implement MRD results in patient care is not a question that will have a simple or universal answer across AML biological groups now or in the near future. Single-cell sequencing technologies are beginning to elucidate the complexities of AML clonal architecture and to provide insight into the functional compartments responsible for disease behavior. Still, these understandings are not yet mature enough to guide surveillance strategies that reliably detect pre-clinical relapse or independently direct patient care [18, 19]. In some cases clinical relapse can occur years after molecular relapse [1]. In some cases, relapsed disease is biologically similar to what was detected at initial diagnosis; in other cases, it can be quite distinct [1, 20].

## Biological foundations for MRD target selection in AML management

### Clinical sources of *in vivo* data for the study of human myeloid diseases

Foundational to any discussion of MRD testing in AML is an understanding of how biologic source material will influence downstream inference. Bone marrow and peripheral blood can both be used for disease monitoring, but the biological equivalence of these compartments remains an area of active study. Blasts can account for up to 5% of healthy bone marrow but should prompt evaluation for malignancy if detected in the peripheral blood, whether or not a patient has been previously diagnosed with a myeloid neoplasm.

A comprehensive review by Butler and colleagues summarizes available data comparing bone marrow- and blood-based MRD assays in AML: peripheral blood sampling captures signals that broadly reflect marrow disease but at reduced sensitivity, while only a handful of cross-validation studies have directly compared the two compartments [21–24]. The kinetics of molecular marker appearance in peripheral blood during relapse are still being elucidated [25]. As such, marrow-based sampling remains the reference standard, though peripheral blood testing offers a less invasive option for longitudinal monitoring.

Because bone marrow biopsies are invasive, AML diagnosis and surveillance in adults generally utilize single-site marrow aspiration. This practice relies on the assumption that the findings from one site represent the entire hematopoietic compartment. Emerging evidence, however, suggests there is spatial heterogeneity of AML clones [26]. Whether multi-site sampling or highly sensitive peripheral assays yield superior predictive power in the era of advanced molecular testing is not yet known [27, 28].

In *de novo* AML, interpretation of diagnostic marrow samples in the absence of pre-diagnosis marrow samples can make it difficult to distinguish acquired somatic mutations that drive leukemia from pre-existing constitutional or CHIP-related variants. Misclassification of benign clonal mutations as markers of residual disease is a recurring concern in MRD assay validation, discussed below.

### Contemporary diagnostic frameworks for myeloid disease management

Modern AML diagnostics are built on classification systems that partition myeloid neoplasms into biologically and prognostically distinct subgroups. Two parallel frameworks are in clinical use: the World Health Organization (WHO) Classification and the International Consensus Classification (ICC) [11, 12, 29, 30]. Both systems incorporate morphologic, cytogenetic, and molecular data to define disease entities and risk categories, superseding the earlier French-American-British system for AML subclassification by morphology. For treatment decisions, these diagnostic tools are commonly paired with a risk stratification schema, such as that proposed by the European LeukemiaNet in 2017 and updated in 2022 [13].

Importantly, the WHO schema recognizes two leukemia precursor states—CHIP and clonal cytopenia of undetermined significance (CCUS)—that are particularly relevant to MRD interpretation (Table 1). Either condition can precede or coexist with AML. CHIP refers to the presence of a hematopoietic clone carrying a mutation associated with malignancy in an otherwise healthy individual, whereas CCUS describes a similar clonal process that is accompanied by one or more otherwise unexplained cytopenias that do not fulfill diagnostic criteria for a myeloid neoplasm. The estimated risk of progression from CHIP or CCUS to overt malignancy parallels that of other premalignant clonal disorders such as monoclonal gammopathy of undetermined significance to multiple myeloma, by design [31]. This risk varies substantially depending on molecular features, such as the variant allele frequency (VAF), the mutations present, and the number of concurrent mutations [32, 33]. For example, for a CCUS with a splicing factor mutation with a VAF > 20%, combined with additional mutations, the 5-year probability of developing a myeloid neoplasm is 95% [34].

**Table 1:** Working definitions for key premalignant and malignant myeloid clonal disorders [11]

	Suggested or formal definition
CHIP*	Detection in bone marrow or peripheral blood of a somatic variant (VAF ≥ 2%), absent associated dysplasia or cytopenia
ARCH	Not well defined in contradistinction to CHIP, may be used when VAF is < 2%
CCUS*	Same as CHIP, but with one or more cytopenia detected
ICUS	Unexplained cytopenia in one or more cell lines for 6 or more months, without detectable somatic mutation, dysplasia, or increase in blast count
MDS*	Can be defined by various molecular findings (e.g., biallelic *TP53* mutation, 5q deletion) in the presence of corresponding blast thresholds, otherwise by dysplasia
AML*	Defined by the presence of defining molecular characteristics, or by threshold blast count, depending on patient-specific factors

*WHO-defined hematologic malignancies. For ARCH and ICUS, no authoritative definitions exist

Abbreviations: *CHIP* clonal hematopoiesis of indeterminate potential; *ARCH* ageing-related clonal hematopoiesis; *CCUS* clonal cytopenia of undetermined significance; *ICUS* idiopathic cytopenia of undetermined significance; *MDS* myelodysplastic syndrome; *VAF* variant allele frequency

The existence of clonal states such as CHIP that can predate AML and persist through successful induction therapy creates a central challenge for MRD testing in AML: not every mutation detected after therapy represents persistent leukemia. Some variants may mark long-standing CHIP clones rather than therapy-resistant AML cells. Distinguishing between these entities requires thoughtful assay design and, when possible, access to germline or pre-leukemic reference material.

### Myeloid clonal dynamics in the evolution and persistence of AML

AML arises within a complex ecosystem of competing hematopoietic clones whose intra- and extracellular features evolve. A single driver mutation may confer different biological behaviors in different hosts, depending on pre-existing molecular and microenvironmental factors. Consequently, it is rarely possible at AML diagnosis to know which subpopulation, whether molecularly or immunophenotypically defined, will dominate or persist following induction therapy. Some genetic or cytogenetic variants that promote leukemic transformation or relapse in one patient’s marrow may be biologically neutral in another’s, and the “second hit” responsible for progression to active disease may not always be identifiable by standard clinical assays. Indeed, within datasets describing ostensibly healthy adults, variants in canonical AML-associated genes—such as *TP53*—have been observed with surprising frequency [35–37]. *ASXL1* mutations, although recurrent in therapy-resistant AML, are too prevalent in CHIP to serve as reliable MRD markers: the 2021 European LeukemiaNet (ELN) MRD Working Party explicitly discourages their use for this purpose [38–41]. As technologies capable of clinical epigenetic profiling become available, assessments of methylation and chromatin states at single-cell resolution may in the future shed light on why different AML cases with the same *ASXL1* mutations, for example, may behave so differently [42, 43].

When considering the biology of the two-hit model in AML, bear in mind that the molecular tools that are today in clinical practice assess patient samples in bulk. Two mutations that co-occur in the same half of a larger population of AML cells will be reported in the same manner as if those mutations occurred mutually exclusive of one another, each in half of the larger population. This reality creates the challenge of colocalization that is common across conventional MRD platforms, but which emerging tools such as single-cell sequencing may help to overcome.

**Fig. 2:**
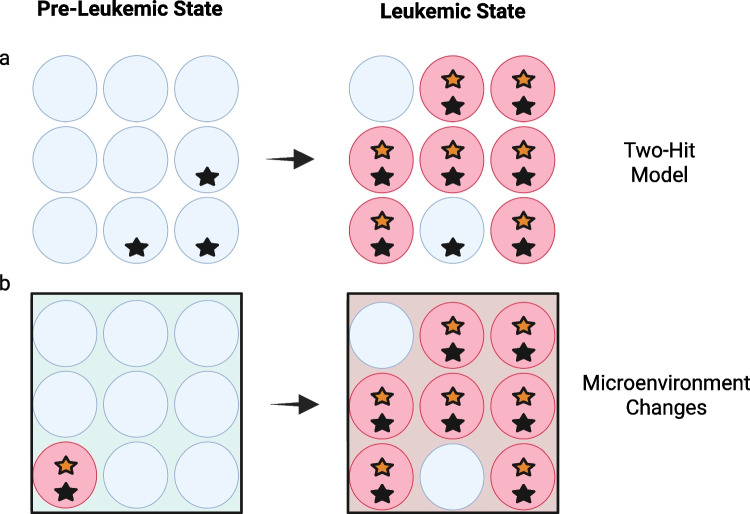
Heuristic patterns of evolution among clones and subclones defined by molecular variants. Blue cells represent non-malignant cells, while red cells represent cells that have become malignant. Stars represent two different molecular variants identified by color. **a** Acquisition of paired mutations provides a sufficient cause for leukemogenesis in the conventional two-hit model. **b** Changes in the microenvironment (represented by background color) allow leukemic expansion of an existing malignant myeloid clone. Created in BioRender. Willis, S. (2025) https://BioRender.com/sezbncg

As an illustration of how challenging it can be to predict the clinical dynamics of AML based on molecular snapshots from marrow or peripheral blood samples, consider a number of possible scenarios, as schematized in Fig. 2 [44, 45]. In one, sequential acquisition of two cooperating mutations results in malignant expansion of a subclone that may or may not fully overtake the progenitor population, sharing certain lineage markers with its non-malignant precursors [42, 46] In another, extrinsic immunologic or metabolic perturbations in the marrow microenvironment allow a previously quiescent clone to expand without acquiring new genetic lesions [47–50].

### Operating characteristics of practical MRD markers in AML

In principle, any molecular variant that can be reproducibly measured could serve as an MRD marker for AML, yet how well a given marker will perform as a predictor of a given outcome can only be established empirically [51]. A driver mutation that serves well as a prognostic biomarker for AML relapse after induction therapy may not reliably predict whether a patient will derive benefit from allo-SCT, for example. As such, MRD positivity might be pursued for two separate, if overlapping, goals. In clinical settings, MRD is pursued as a risk factor for a given clinical outcome. In basic research settings, there may be a greater interest in defining biologically relevant markers of clonal persistence after treatment.

Across hematologic malignancies, MRD results are today most typically applied in clinical decision-making through frequentist statistical frameworks that relate relapse risk to population-level probabilities defined at binarized assay cut-offs. Optimizing such thresholds is especially difficult for low-frequency events, given that such approaches assume that true relapse risk is identical in small study cohorts and the patient populations they sample [52, 53].

Because consensus does not yet exist in the field for how raw MRD data should be applied and assay cut-offs assigned, it is essential that contemporary studies report sufficiently rich data to support re-analysis as standards shift. A good example of transparent reporting is the German–Austrian AML Study Group 16-10 trial that shows a “dose dependent” risk of relapse with the application of different thresholds for binarization of MRD positivity among patients with *FLT3-ITD* AML [54]. Such reporting also allows study results to be better personalized for application to individual patients, whose risk for a clinical outcome of interest can be based on the stratum of quantitative MRD into which their personal testing results fall.

Looking forward, exploration and adoption of Bayesian analysis might precipitate a paradigm shift in how AML MRD data are used to guide patient care decisions, as addressed in a manuscript distributed before publication by Cremaschi and colleagues [55]. Using a Bayesian approach, a patient’s estimated risk of AML relapse can be better personalized by applying MRD testing results in the context of individual disease and treatment factors. Rather than estimating relapse risk based on positive *versus* negative MRD results, MRD results are used to refine a risk of relapse previously estimated based on clinical factors that can vary among individuals within study populations [56, 57].

## Established tools for MRD integration and investigation in AML

Although MRD testing and MRD-adapted treatment approaches are established in several hematologic malignancies, their integration into the clinical management of AML has progressed in a more measured manner. While a few molecular tools have received approval from the U.S. Food and Drug Administration for diagnostic use in AML, none are yet approved specifically for MRD measurement. Moreover, no MRD-based paradigms for accelerated drug approval or treatment adaptation have yet emerged in AML, unlike the regulatory frameworks that exist for multiple myeloma [58].

Current data most strongly support the use of MRD testing to guide management of core-binding factor (CBF) AML, *NPM1*-mutated AML, and *FLT3*-mutated AML, where disease-specific molecular markers are well defined and assays are relatively mature [59, 60]. In these contexts, MRD negativity following two cycles of intensive therapy and/or at the end of consolidation is consistently associated with improved relapse-free and overall survival. Conversely, MRD persistence or re-emergence often heralds relapse and can prompt consideration of treatment intensification or allo-SCT in a patient for whom transplantation was initially deferred.

Professional society guidelines addressing MRD in AML generally fall into two broad categories: those providing expert consensus on the integration of existing MRD tools based on published data, and those establishing technical standards to guide assay development and reporting. The most influential examples in the first category are the recommendations published by the National Comprehensive Cancer Network (NCCN) and the ELN [12, 14]. Each outlines which AML subtypes are most amenable to MRD assessment, when MRD testing should be performed, and how MRD results might influence treatment decisions.

Both NCCN and ELN guidelines recommend incorporating genetic MRD assessment with multicolor flow cytometry (MFC) in parallel, though interpretation frameworks for flow-based MRD assays are not well standardized (Table 2) [13, 14]. Molecular MRD monitoring in AML is currently based largely on qPCR assays for specific gene targets—*NPM1* mutations, *RUNX1-RUNX1T1* translocations, and *CBFB-MYH11* translocations. NGS methods are under active investigation as tools capable of detecting multiple variants simultaneously and with greater sensitivity, albeit still in bulk [63, 64]. NGS platforms are not yet endorsed for routine MRD monitoring outside of research protocols, largely because of ongoing concerns regarding inter-laboratory variability and uncertainty around analytical thresholds.

**Table 2:** Selected society recommendations for the development and implementation of measurable residual disease testing in the management of acute myeloid leukemia [38, 61, 62]

Professional society	Year	Central findings and recommendations
NHS Chief Scientific Officer’s Knowledge Transfer Partnership	2024	Technical recommendations • Use *ABL1* as a reference for qPCR-based assays to allow inter-laboratory standardization • Higher order standardization approaches for traceable normalization to reference materials should be implemented in MRD development
European LeukemiaNet MRD Working Party	2021	• Nucleic acid sequencing assays ◦ Use only 5 mL of BM aspirate for molecular MRD assessment ◦ For patients with classical translocations, qPCR or dPCR should be used to track MRD status ◦ Data are insufficient to support monitoring by NGS alone ◦ Molecular MRD testing approaches should be designed and validated for a limit of detection of 10^−3^ or lower ◦ Germline mutations and mutations commonly seen in age-related clonal hematopoiesis (e.g., DNMT3A, TET2, and ASXL1) should be excluded from biomarker candidacy ◦ MRD test positivity by qPCR is defined as a cycling threshold < 40 in at least 2 of 3 replicates (with at least 10,000 copies, and optimally ≥ 30,000 copies of the housekeeping gene measured) • Flow cytometry assays ◦ Both LAIP and DfN approaches should be used simultaneously to optimize capture of aberrant clones ◦ Use first-pull marrow samples and analyze within 3 days of collection ◦ Assess samples for peripheral blood contamination ◦ Analyze at least 5 × 10^5^ CD45-positive cells and at least 100 viable blast cells before concluding MRD negativity ◦ Marker sets should minimally include CD34, CD117, CD45, CD33, CD13, CD56, CD7, and HLA-DR ◦ Pathology reports should recommend repeat testing in 2–4 weeks when immunophenotypic changes may manifest due to timing (e.g., regenerating marrow after treatment) ◦ Test positivity is defined as ≥ 0.1% of CD45-positive cells with the target immunophenotype
Food and Drug Administration	2020	• MRD should be determined from bone marrow at the time point of a complete remission with blood count recovery • To use a selected marker for MRD, data should be provided showing the marker is specific to the leukemia and not underlying clonal hematopoiesis; the false-negative rate should also be described

Abbreviations: *DfN* different-from-normal; *LAIP* leukemia-associated immunophenotype; *dPCR* digital PCR

Notable among technical documents for AML protocol development and refinement are the 2021 ELN recommendations for MRD testing in AML and the 2024 guidance from the UK National Health Service (NHS), both of which address sample collection, processing, minimal cell count requirements, data analysis, and post-analytical quality control [38, 61, 65]. These frameworks emphasize that MRD assays must be validated with clear communication of lower limit of detection (LLOD), lower limit of quantification (LLOQ), and associated uncertainty near assay thresholds (Table 3).

**Table 3:** Selected test characteristics for measurable residual disease assays [65–68]

Term	Definition	Notes on significance
Nucleic acid sequencing		
Read depth	How many times a genetic locus is read in each sequencing run	Greater read depth allows greater accuracy of base pair calling. Rarer variants need to be read at greater depths to be called accurately. Generally reported as a single value across the entire target genetic area
Read coverage	The portion of intended bases (e.g., whole genome *versus* whole exome) that have been read at the goal depth	Long-read tools may offer better coverage
Quality score *(Q)*	*Q* = −10 *log* Pr(inaccurate read)	Quality scores require a computational method to estimate the chance of a given read base being inaccurate and are used to exclude reads in sections of the genome where they are considered unreliable. Methods and thresholds for exclusion vary
Flow cytometry		
Level of background (LOB)	(Mean frequency of IP of interest in healthy samples in relevant clinic setting) + n*(SD of that mean)	*n* is selected based on the tolerance for imprecision and the statistical distribution used to represent immunophenotype representations (e.g., standard normal approximation, Poisson distribution). Estimating SD requires multiple replicates of healthy sample measurement
Limit of detection (LOD)	LOB + n*(SD)	*n* as above, note that the distribution for LOD may be different than for LOB. In this case SD is for the distribution of IP of malignant cells
Lower limit of quantification (LLOQ)	LOD + CV	CV selected based on tolerance for reliability, based on sampling distribution

Abbreviations: *CV* coefficient of variation; *IP* immunophenotype; *Pr* probability; *SD* standard deviation

In addition to the aforementioned sources, readers are also directed to forthcoming updates from the ELN-DAVID MRD Working Party, which will include both clinical and technical consensus recommendations [69].

Understanding what each method measures, its practical limits of detection, and what varieties of residual disease it might fail to capture due to pre- or post-analytic peculiarities is essential for the meaningful integration of MRD testing into AML management. To that end, the following section reviews each major MRD detection approach—cytogenetic, genetic, and immunophenotypic, each with discussions of the strengths, weaknesses, and data supporting use in specific clinical settings.

## Contemporary platforms for modern MRD measurement in AML

Genetic and cytogenetic assays form the historical foundation of AML diagnostics, and many of the same platforms are now being explored for MRD detection. These tools differ not only in their analytical sensitivity but also in what aspects of disease biology they can capture. The practical sensitivities of these assays span several orders of magnitude—from approximately one malignant cell in ten analyzed for conventional cytogenetics to one in 100,000 or more for next-generation or multimodal molecular assays. All of the tools that are today available for clinical use are performed by analyzing samples in bulk, as discussed above.

### Cytogenetic approaches

Cytogenetic methods remain central to the initial diagnosis and risk stratification of AML, and their role in MRD monitoring has been explored. Conventional karyotyping assesses metaphase chromosome spreads from cultured marrow cells and is capable of detecting large structural and numerical chromosomal abnormalities. However, because only a limited number of metaphases—typically around 20—are examined in a standard analysis, the method lacks sensitivity for detecting small residual populations or low-frequency clones. Culture-based expansion introduces further bias, as not all clones proliferate equally well *in vitro*. Nevertheless, isolated reports have demonstrated the prognostic value of cytogenetic persistence in certain AML subtypes [70].

To improve on the sensitivity of traditional karyotyping, FISH has been widely adopted for the detection of known chromosomal translocations and copy number changes. FISH utilizes fluorescent probes targeting specific genomic regions and can identify aberrations at the single-cell level, allowing detection of abnormal clones at roughly ten times greater sensitivity than karyotyping [71]. However, FISH is inherently a targeted technique and cannot detect novel or unexpected abnormalities for which probes are not supplied. It is most valuable for confirming the clearance or persistence of known genetic lesions, such as those involving the *RUNX1-RUNX1T1* or *CBFB-MYH11* fusions.

More recently, chromosomal microarray analysis (CMA) and optical genome mapping (OGM) have provided additional tools for genome-wide surveys for copy number and structural variants. CMA offers the additional ability to detect copy-neutral loss of heterozygosity, which neither karyotyping nor FISH can identify [2, 72]. Despite this advantage, CMA assays have a relatively high LLOD, making them generally less suitable for MRD assessment [72]. OGM, on the other hand, is an emerging tool that allows for rapid detection of cytogenetic changes across the genome without requiring cell culture, and early studies suggest it may be a powerful tool in both diagnostic and MRD applications.

### PCR-based genetic approaches

PCR-based assays offer substantially improved analytical sensitivity compared with cytogenetic methods and remain the backbone of MRD detection in AML when suitable targets are available. Traditional real-time quantitative polymerase chain reaction (qPCR) enables detection and quantification of pre-defined transcripts or mutations with a sensitivity typically around one in one thousand (10^−4^). However, its reliance on the construction of standard curves based on serial samples and its susceptibility to inhibitors that can introduce quantification variability are especially problematic in the MRD setting.

Within the family of conventional PCR, droplet digital PCR (ddPCR) offers potential solutions to these shortcomings. Synthetic reactions in ddPCR are partitioned into thousands of droplets, allowing amplification to occur in parallel microreactions. This configuration enables absolute quantification without the need for standard curves and minimizes the effect of inhibitory substances. The resulting sensitivity improvement—approximately one log (tenfold) over standard qPCR—permits reliable detection of low-frequency variants in a wide variety of sample types [73]. While ddPCR assays have not yet received regulatory approval for AML MRD monitoring, active investigations, particularly for *FLT3* and other recurrent mutations, show encouraging feasibility [74, 75].

### Next-generation sequencing approaches

NGS technologies have already transformed AML diagnosis and now represent some of the most promising avenues for MRD assay development. Unlike classical PCR, which requires predefinition of specific targets for measurement, NGS allows for simultaneous interrogation of many loci in parallel, and for the detection of both known and novel variants. Implementation of NGS for MRD remains technically complex, given the numerous analytical factors that must be tuned to optimize test performance. Note that there is not a single definition for what constitutes a next-generation sequencing tool—NGS tools vary widely in technical approach, availability, cost, and practicality for incorporation into MRD workflows.

Relevant NGS tools fall broadly into two categories: (1) those that use sequencing by synthesis (analogous to PCR) and (2) those that rely on alternative sequencing chemistries. Within either category, laboratories may opt for short-read sequencing (hundreds of base pairs read per nucleic acid fragment) or long-read sequencing (thousands to tens of thousands of base pairs). Long-read platforms might be advantageous in some MRD settings due to their avoidance of systematic errors associated with amplification, particularly in GC-rich or repetitive regions such as the *FLT3-ITD* locus, and because they can directly map structural variants missed by short-read approaches [76, 77]. Short-read methods remain more widely used and studied for the time being due to their lower cost and higher per-base accuracy.

Across NGS platforms, depth of sequencing provides an important tool for comparability (Table 3). Greater read depth enhances the detection of low-frequency variants but increases cost and computational burden. One solution that might allow balancing of broad reads across the genome with sufficient depth in regions of interest is reflex testing to a more sensitive assay such as qPCR when NGS detects a variant between the LLOD and LLOQ, for example.

As sequencing costs decline and bioinformatic pipelines become more standardized, NGS is expected to assume an increasingly prominent role in MRD monitoring, particularly when combined with positive cell selection strategies that enrich for malignant populations prior to sequencing, as explored in further detail below [78].

### Immunophenotypic approaches

While genetic assays provide powerful information about molecular persistence, immunophenotypic (IP) tools remain indispensable for characterizing residual disease at the cellular level. Current professional society recommendations advise that these approaches be used in parallel with genetic and cytogenetic tools, though there is increasing interest in the sequential use of IP with molecular assessments.

MFC quantifies the expression of surface and intracellular markers across large numbers of cells, enabling the identification of rare aberrant populations. Diagnostic panels typically employ six to ten fluorescent channels, though newer spectral flow cytometers can analyze forty or more. Depending on assay design, marker stability, and operator experience, modern assays have effective sensitivities of approximately 10^−3^ to 10^−4^. Major strengths of MFC in clinical diagnosis and MRD testing are speed, accessibility, and the ability to provide real-time information about residual abnormal populations. Major limitations relating to disease biology include false positives (e.g., regenerating marrow) and false negatives in hypocellular specimens. To improve reproducibility, ELN guidelines emphasize the inclusion of LLOD and LLOQ values in all MFC reports and recommend detailed interpretive commentary by experienced hematopathologists [79–83]. Still, adoption of IP tools for MRD measurement will demand standardization of approaches across institutions, where different combinations of instrumentation, reagents, and subjective reviewer expertise will otherwise generate different results.

As explored above, AML is not a disease for which a single or series of widely expressed surface markers can be used as pathognomonic markers of disease. In this setting, two elegant approaches have been established to measure MRD signal in AML *via* MFC. Given their individual shortcomings, the ELN now recommends combining both approaches, leukemia-associated immunophenotype (LAIP) and different-from-normal (DfN), for comprehensive assessment [84–89]. The LAIP approach monitors for persistence of patient-specific abnormal marker patterns identified at diagnosis, while the DfN approach identifies cells whose marker combinations fall outside normal hematopoietic patterns [38].

While LAIP testing assumes immunophenotypic stability and requires baseline testing for stability, it is less reliant on the assumption that certain myeloblast populations can be defined as malignant in a patient-agnostic manner. No standard panel has been universally adopted to probe for either MRD pattern, though guidance from the ELN recommends CD34, CD117, CD45, CD33, CD13, CD56, CD7, and HLA-DR be included in panels designed at the institutional level [38]. Whether local or commercial assays are used, simulation and validation runs are needed for each package of markers that is to be clinically implemented [90]. When considering the pairing of LAIP and DfN assays in this setting, note that diagnostics in general can be combined using hierarchical, reflex, or parallel testing approaches for development of compound MRD tools, but that the statistical considerations involved in anticipating overall performance based on the characteristics of constituent elements are not trivial [38, 88, 89, 91].

## Emerging multimodal approaches

Recognizing the limitations of the individual assays and platforms that have been presented, a number of emerging multimodal techniques are becoming important considerations for those seeking to leverage the strengths of multiple assay types at once for combined MRD performance.

One such promising development is Flow-FISH, which combines the probe-based specificity of FISH with the high-throughput cell counting of flow cytometry. This hybrid approach allows the detection of low-frequency events by analyzing tens of thousands of cells, rather than the few hundred examined in traditional FISH. Although Flow-FISH has not yet been applied to AML MRD, feasibility has been demonstrated in multiple myeloma [92, 93].

Combining MFC and cell sorting with molecular diagnostics are platforms that permit positive or negative cell selection prior to NGS, for example, to enrich for cells with aberrant immunophenotypes. This integration of MFC and molecular methods enhances sensitivity and allows some localization of genetic variants to malignant populations, improving the signal-to-noise ratio and clarifying whether detected mutations arise from AML or from CHIP-related clones [94–97].

Finally, single-cell sequencing (SCS) represents a powerful frontier for MRD detection, capable of assessing genotype and phenotype within individual cells. Generating barcoded nucleic acid reads that can be traced to specific source cells as defined by surface markers, single-cell DNA sequencing can theoretically detect one leukemic cell among 10,000, corresponding to a sensitivity near 10^−4^ to 10^−5^, though gene dropout and sampling constraints can limit real-world performance [98, 99]. Proof-of-principle applications of SCS in AML have already demonstrated its potential to map clonal hierarchies and identify relapse-driving subpopulations, but these have not yet entered the clinic [100]. As with other multimodal tools, SCS offers results that will advance our understanding of how AML functions, even as they guide rational and personalized patient care.

## Conclusion

Taken together, the preceding sections illustrate both the promise and difficulty of MRD integration into AML management. The field now stands at an intersection of clinical evidence, technical innovation, and biological nuance. Data from large academic and cooperative studies confirm that MRD negativity, as clinically evaluable, correlates with improved survival in some important patient subsets. Persistent gaps in assay standardization, interpretive frameworks, and biological validation constrain routine clinical use.

One of the foremost challenges in AML MRD assessment lies in distinguishing true leukemic persistence from benign clonal hematopoiesis. Age-related clonal expansions, including CHIP and CCUS, may harbor mutations identical to those driving AML. This overlap can lead to false-positive MRD results if detected variants are misinterpreted as residual leukemia. Conversely, reliance on narrow mutation panels can yield false negatives for relapse surveillance, particularly when relapse arises from subclones carrying different genetic lesions than those present at diagnosis.

Bulk sequencing methods, though powerful, provide limited spatial and clonal resolution. They cannot determine whether multiple mutations coexist within the same cell or among distinct subclones. Emerging single-cell and positive-selection approaches may mitigate this limitation but remain labor- and cost-intensive. The performance of flow cytometric MRD detection depends on assumptions about IP repertoires, yet phenotypic drift following therapy is common. Shifts in antigen expression can obscure residual leukemic populations or generate false signals from regenerating marrow.

Institutional differences in assay sensitivity and reporting practices further complicate MRD implementation based on literature reporting. Even well-validated tests only operate within quantifiable error margins defined by their LLOD and LLOQ. Results near these thresholds must be interpreted cautiously, particularly when minor changes may influence major treatment decisions. Moreover, MRD performance depends heavily on sample quality and timing. Hypocellular post-therapy marrows, hemodilute aspirates, or delayed sample processing can all reduce analytical sensitivity.

Future progress in AML MRD research will depend on the integration of complementary detection modalities. Multimodal strategies that combine flow cytometry, PCR, and sequencing—each with distinct strengths—can improve both sensitivity and specificity. As tools such as Flow-FISH, flow-based cell enrichment before sequencing, and single-cell multi-omic profiling mature, they are expected not only to refine MRD detection but also to enhance understanding of AML clonal biology and therapy resistance. The “leukemic stem cells” that are theorized to compose the self-renewing and therapy-resistant compartment in AML are not yet understood well enough for either reliable characterization or prediction of their behavior [101]. Late relapses are rare but do occur in AML, and in case series have been reported to often harbor mutations characteristic of previously treated disease, suggesting a dormancy state that future MRD assays will aim to detect [102, 103].

Even using the MRD detection tools of the future, how well an MRD-informed treatment approach will serve a patient is an empiric question that will depend on how and when such a marker is assessed in each context. Some markers might function well prognostically and others predictively, sometimes as a result of non-intuitive statistical phenomena. To optimize and expand the use of the genetic, cytogenetic, epigenetic, immunophenotypic, and combined-modality tools now available in the field will require ongoing collaboration between patient-facing clinicians and laboratory investigators in order to implement more powerful—and more sophisticated—approaches to AML monitoring.

## Data Availability

No datasets were generated or analysed during the current study.
